# Infectivity and Morphology of Bovine Coronavirus Inactivated In Vitro by Cationic Photosensitizers

**DOI:** 10.3390/v14051053

**Published:** 2022-05-15

**Authors:** Vladimir Zhukhovitsky, Natalia Shevlyagina, Margarita Zubasheva, Leonid Russu, Vladimir Gushchin, Gennady Meerovich, Marina Strakhovskaya

**Affiliations:** 1 Gamaleya National Research Centre for Epidemiology and Microbiology, Moscow 123098, Russia; zhukhovitsky@rambler.ru (V.Z.); nataly-123@list.ru (N.S.); mzubasheva@mail.ru (M.Z.); plano77@bk.ru (L.R.); wowaniada@gmail.com (V.G.); 2 Russian Medical Academy of Continuing Professional Education “RMANPO”, Moscow 125993, Russia; 3 Prokhorov General Physics Institute of the Russian Academy of Sciences, Moscow 119991, Russia; gennadymeerovich@gmail.com; 4 Institute for Physics and Engineering in Biomedicine, National Research Nuclear University “MEPHI”, Moscow 115409, Russia; 5 Faculty of Biology, Lomonosov Moscow State University, Moscow 119234, Russia; 6 Federal Scientific and Clinical Center of Specialized Types of Medical Care and Medical Technologies of the Federal Medical and Biological Agency of Russia, Moscow 115682, Russia

**Keywords:** bovine coronavirus, glycoprotein, photosensitizer, photodynamic inactivation, octakis(cholinyl)zinc phthalocyanine, methylene blue, transmission electron microscopy

## Abstract

Bovine coronaviruses (BCoVs), which cause gastrointestinal and respiratory diseases in cattle, and are genetically related to the human coronavirus HCoV-OC43, which is responsible for up to 10% of common colds, attract increased attention. We applied the method of photodynamic inactivation with cationic photosensitizers (PSs) to reduce the titers of BCoV and studied the morphological structure of viral particles under various modes of photodynamic exposure. The samples of virus containing liquid with an initial virus titer of 5 Log_10_ TCID50/mL were incubated with methylene blue (MB) or octakis(cholinyl)zinc phthalocyanine (Zn-PcChol_8+_) at concentrations of 1–5 μM for 10 min in the dark at room temperature. After incubation, samples were irradiated with LED (emission with maximum at 663 nm for MB or at 686 nm for Zn-PcChol_8+_) with light doses of 1.5 or 4 J/cm^2^. Next, the irradiation titrated virus containing liquid was studied using negative staining transmission electron microscopy. MB and Zn-PcChol_8+_ at concentrations of 1–5 μM, in combination with red light from LED sources in the low doses of 1.5–4.0 J/cm^2^, led to a decrease in BCoV titers by at least four orders of magnitude from the initial titer 5 Log_10_ TCID50/mL. Morphological changes in photodamaged BCoVs with increasing PS concentrations were loss of spikes, change in shape, decreased size of virus particles, destruction of the envelope, and complete disintegration of viruses. BCoV has been found to be sensitive to MB, which is the well-known approved drug, even in the absence of light.

## 1. Introduction

Coronaviruses (CoVs) are enveloped viruses with single-strand positive-sense RNA genomes that belong to one of the four genera, Alpha-, Beta-, Gamma-, and Delta coronavirus, in the Coronaviridae family, of the order Nidovirale. The hosts of the 45 identified CoVs species are vertebrates, birds, and mammals [1,2]. Coronaviruses affecting farm animals such as bovine coronavirus, porcine haemagglutinating encephalomyelitis virus, porcine transmissible gastroenteritis virus, hen infectious bronchitis virus (IBV), and others, cause serious economic damage [2,3,4,5]. Farm animals can also serve as reservoirs and intermediate hosts of coronaviruses, including their mutated forms, enabling participation in interspecies transmission [6]. In this respect, bovine coronaviruses are of great interest, causing gastrointestinal and respiratory diseases in cattle, and demonstrating a close relationship with the human coronavirus HCoV-OC43, which causes up to 10% of common colds. As it has been suggested, around 1890 BCoV were able to cross the species barrier and become capable of infecting humans, leading to the emergence of a new type of human coronavirus HCoV-OC43 [7]. The fact that BCoV-related coronavirus has been isolated from human diarrhea samples indicates its importance to public health [4].

BCoVs range in diameter from 65 to 210 nm and have a crown formed by a long 20 nm homotrimeric spike (S), and additional short 8-nm homodimeric hemagglutinin-esterase (HE) surface glycoproteins [8]. The most abundant (1000–2000 molecules per virion) envelope protein is the membrane M protein with a short ectodomain [9]. Eight M dimers accommodate up to four nucleoproteins and one trimeric S-protein [10], and the number of spikes is around 25 ± 10 trimers per virion [11]. In addition to S-proteins, Betacoronaviruses of lineage A use HEs as a second protein to initiate infection of host cells [12]. The HE of BCV is the 140-kDa protein dimer of the 65-kDa glycosylated subunits [13]. Photodynamic inactivation (PDI) is an established method to combat pathogenic viruses, bacteria, fungi, and protozoa [14,15,16,17]. It utilizes the destructive effects of reactive oxygen species produced by photoactivated photosensitizers (PSs). PSs of different chemical classes (tetrapyrrole derivatives, phenothiazines, fullerenes, etc.) in combination with appropriate light, reduce titers of viruses and bacteriophages with the efficacy summarized in [18,19]. PSs, which induce the formation of singlet oxygen, appear to be promising antiviral agents, especially against enveloped viruses with their readily oxidized surface proteins and lipid membrane.

Large, single-strand RNA bearing enveloped coronaviruses are potential sensitive targets to reactive oxygen species generated by various PSs [20,21]. Recently, we have shown the existence of a negatively charged binding site for cationic PSs at the connection of the S-protein stalk and the head adjacent to the HR2 domain, which is common to the S-proteins of SARS-CoV, SARS-CoV-2, and MERS-CoV [22], all of which belong to the same with BCoV genus Betacoronavirus. One of these water-soluble cationic PSs, octakis (cholinyl) zinc phthalocyanine (Zn-PcChol_8+_), in combination with far red light irradiation, was able to completely destroy the infectivity of SARS-CoV-2 in suspensions with the initial titer 4.75–5.00 Log_10_ TCID50/mL [23]. Another enveloped avian H5N8 influenza virus was also susceptible to photodynamic inactivation with Zn-PcChol_8+_. Transmission electron microscopy (TEM) revealed that H5N8 virus membranes kept their structural integrity but lost their surface glycoproteins [24]. The result of the mild photodynamic treatment was the detachment of the influenza virus surface glycoproteins and the loss of infectivity by “bald” virions; however, at a higher Zn-PcChol_8+_ concentration, the envelope was totally destroyed [24]. Another cationic PS methylene blue (MB), in the range 1–10 µg/mL with irradiation 662 nm, fully protected Vero E6 cells from infection with 4 Log_10_TCID50 of SARS-CoV-2, and partly protected them from 5 Log_10_ TCID50 of SARS-CoV-2 [25]. In contrast to Zn-PcChol_8+_, with very low “dark” antiviral activity against SARS-CoV-2 [23], MB 50 % inhibitory concentration (IC50) against 10^2^ TCID50 of SARS-CoV-2 was found to be 0.22 μg/mL [25]—that is about 0.7 µM. In our model experiments [22], MB was able to penetrate inside the pocket of the S-protein head formed by the transition of the receptor binding domain (RBD) into the “open” state. The binding of MB to the RBD domain of the model S-protein [22] corresponds to the dark antiviral effect of this PS, which is capable of reducing SARS-CoV-2 infectivity in a Vero E6 cell culture [25]. The existence of the binding site for cationic PSs, which is common to the S-proteins of SARS-CoV, SARS-CoV-2, and MERS-CoV, creates prospects for the wide use of this type of PSs to combat the spread of coronaviruses.

In this study, for the first time, we applied the method of photodynamic inactivation with a cationic Zn-phthalocyanine derivative to reduce the titers of BCoV, and we studied the morphological structure of viral particles under various modes of photodynamic exposure.

## 2. Materials and Methods

### 2.1. Virus Propagation and Titration

In this work, we used BCoV, which is considered as a model coronavirus for the study of SARS-CoV-2 [19,20], as well as the substantive viral object. BCoV (strain CV-90) was obtained from Russian Federation National Virus Collection (D.I. Ivanovsky Institute of Virology Division of N.F. Gamaleya National Research Center of Epidemiology and Microbiology of the Ministry of Health of the Russian Federation, Moscow). All experiments were performed according to the standard protocols at the BSL 3 Laboratory and reference center for coronavirus infection (Gamaleya National Research Center of Epidemiology and Microbiology, Moscow, Russia). Work with pathogenic microorganisms was regulated by the rules for working with biological agents of III-IV pathogenicity groups (Sanitary and epidemiological rules SP 1.3.3118-13).

Stocks of BCoV were obtained during acute infection of MDBK (bovine kidney) cells, which were cultured in a complete DMEM growth medium containing 5% fetal bovine serum (FBS), L-glutamine (4 mM), and a mixture of antibiotics penicillin/streptomycin (100 IU/mL; 100 µg/mL).

Tenfold serial dilutions of virus containing liquid (up to 10^−6^) with 0.9 mL of DMEM medium were used for titration on MDBK cells. Titration was performed in quadruplicate. The day before the experiment, the MDBK cell culture was seeded into 96-well plates at a concentration of 2.5 × 10^4^ cells/well. The growth medium was removed from the wells with the cell monolayer and the prepared dilutions of virus containing liquid were added (200 µL per well). After that, plates were incubated for 5 days at 37 °C and 98% humidity in an atmosphere with 5% CO_2_. A virus-induced cytopathic effect was detected, and virus titers were determined using the 50% tissue culture infectious dose (TCID50) per ml, in accordance with [26]. In all experiments, cells that were not infected with the virus were included as controls.

### 2.2. Photodynamic Inactivation

**Figure 1:**
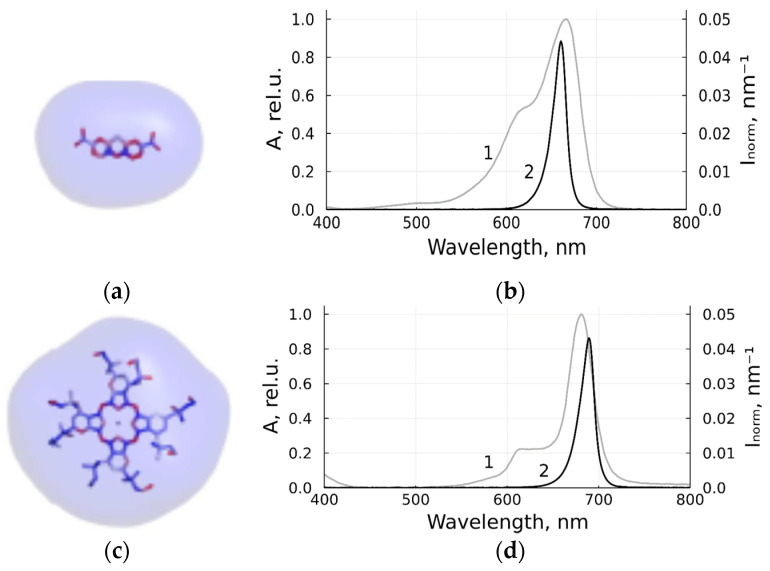
Cationic PSs used in this study and LEDs for their excitation: monocationic MB (**a**,**b**), octacationic Zn-PcChol_8+_ (**c**,**d**); the stick models of PSs with equipotential electrostatic surfaces colored by red (−7 mV) and blue (+7 mV) in accordance with [22] (**a**,**c**); normalized spectral contours of the absorption bands of PSs (1) and normalized emission spectra of LED sources (2) in accordance with [27] (**b**,**d**).

PSs used in this study were monocationic 3,7-bis-(Dimethylamino)-phenazathionium chloride C16H18ClN3S (methylene blue, Samaramedprom, Samara, Russia) and octacationic octakis(cholinyl)zinc phthalocyanine (Zn-PcChol_8+_, Organic Intermediates and Dyes Institute, Moscow, Russia). The stick models with equipotential electrostatic surfaces of MB, with a total charge of +1, and Zn-PcChol_8+_ with a total charge of +8, are shown in Figure 1, in accordance with our previous study [22,27].

Photodynamic inactivation of BCoV was induced with irradiation of light sources based on LEDs. Light sources of suitable wavelengths required for PS excitation were designated based on single high-power LEDs [28], using LC-1DR-G30 for the spectral range of 660–665 nm, and LC-1IR1-G42 for 680–690 nm manufactured by Shenzhen Cai Mingzhu Co, LTD (Shenzhen, China). The optical system of the device projected the image of LED onto the surface of the sensitized layer in the optical cell that contained sensitized virus containing liquid and provided uniform photodynamic inactivation in the treated sample. Figure 1 shows the normalized spectral contours A(λ) of the absorption bands of studied PSs (the absorption spectrum at low concentration divided by its maximum value) and the normalized emission spectra Inorm(λ) of LEDs (light intensity spectral density divided by the integral intensity). The developed sources provide a power density of about 21 mW/cm^2^.

After incubation with PS (1–5 μM) in square 1 × 1 cm^2^ plastic optical cells for 10 min in the dark at room temperature, virus containing samples (400 µL) were irradiated in the same cells for 72 or 190 s, at light doses of 1.5 or 4 J/cm^2^, respectively. The experiments were carried out in triplicate. After irradiation, 0.2 mL of VCL were used for electron microscopy and 0.2 mL were used for titration. Samples incubated with PS (1–5 μM) under the same conditions, but without further irradiation, served as the “dark control”. Control virus containing samples were incubated during the experiment without PS in the dark at room temperature.

### 2.3. Transmission Electron Microscopy with Negative Contrast

A drop of the sample suspension, taken with a Pasteur pipette, was placed on the surface of a formvar coated and carbon evaporated 200 Mesh copper grid (EMS, Hatfield, PA, USA) for 1 min. Suspension excess was removed with filter paper. Next, a drop of 2% uranyl acetate solution (Serva Electrophoresis, GmbH, Heidelberg, Germany) was applied to the surface of the test sample for 1 min. The excess of the latter was also removed with filter paper. Samples were dried for 10 min at room temperature and then analyzed in a “JEM 2100Plus” transmission electron microscope (JEOL, Akishima, Japan) at an accelerating voltage of 160 kV. Images were captured by a Nanosprt5 camera (AMT, USA), exposure: 300 ms × 15 std frames.

## 3. Results

### 3.1. Antiviral Activity of MB and Zn-PcChol_8+_ without Irradiation

**Figure 2:**
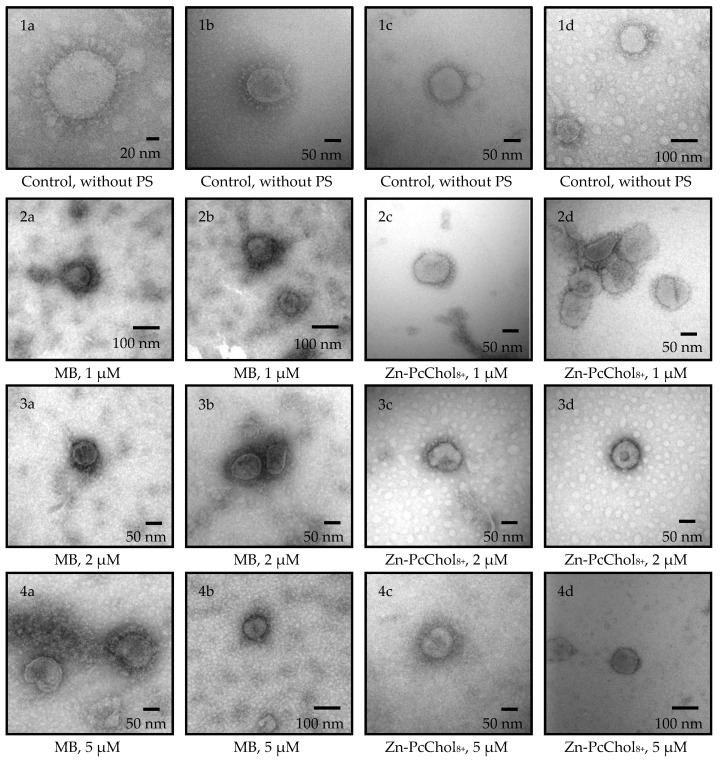
Electron micrographs of BCoV negatively stained with 2% uranyl acetate after 10 min incubation in the dark without PS (control), or with different concentrations of MB or Zn-PcChol_8+_.

The initial titer of the virus, propagated and determined using monolayers of MDBK cells, was 5 Log_10_ TCID50/mL. Negatively stained BCoVs had a pleomorphic or roughly circular shape with a diameter of 120 to 160 nm, including a double fringe or corona formed by two types of projections (Figure 2). The longer ones, which are S-proteins (spikes), protrude beyond the membrane-surrounded part of the virions by about 20–22 nm. The shorter ones, representing hemagglutinin esterase (HE), are more than twice as short. Thus, on electron micrographs, one can see the morphology characteristics of BCoV [29,30].

Recently, we showed that both dyes, monocationic MB and octacationic Zn-PcChol_8+_, possess an affinity for negatively charged regions of coronavirus spikes, the main region of which is located at the connection of the S-protein stalk and the head on the linker between the HR1 and HR2 domains, adjacent to HR2 [22]. Incubation of BCoV for 10 min using these dyes leads to a deterioration in the resolution of spikes (Figure 2) during electron microscopy, negatively contrasting with uranyl acetate. A decrease in the size and disruption of the form of virus particles was typical after treatment with MB and Zn-PcChol_8+_ without irradiation, especially at concentrations of 2–5 µM.

**Figure 3:**
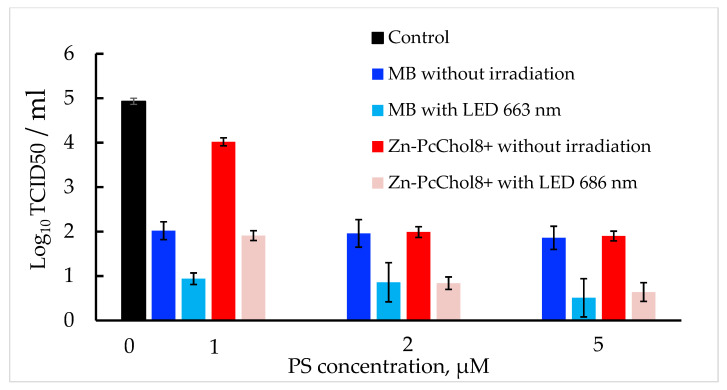
BCoV titers without PSs and irradiation (control), preincubated at 10 min, with different concentrations of PSs without further irradiation, or irradiated with LED 663 nm for MB, or 686 nm for Zn-PcChol_8+_ photoactivation, in a dose of 4.0 J/cm^2^.

Both PSs, even in the absence of irradiation, decreased BCoV titers (Figure 3). The most pronounced “dark” antiviral activity towards BCoV was exhibited by MB, which at a concentration of 1 μM, caused a decrease in the initial titer of 5 Log_10_ TCID50/mL by three orders of magnitude at a concentration of 1 μM. This coincides with the previously found high “dark” toxicity of MB, with IC50 0.22 μg/mL (about 0.7 µM), against 2 Log_10_ TCID50 of pandemic SARS-CoV-2 [25]. Zn-PcChol_8+_, at a concentration of 1 μM, caused a significantly lower reduction in BCoV titer compared with MB, but it had a similar “dark” antiviral activity at concentrations of 2–5 µM. This differs from SARS-CoV-2 [23], as Zn-PcChol_8+_ in the concentration range of 1–5 μM showed practically no toxicity in the absence of irradiation against the pandemic coronavirus.

### 3.2. Photodynamic Inactivation

**Figure 4:**
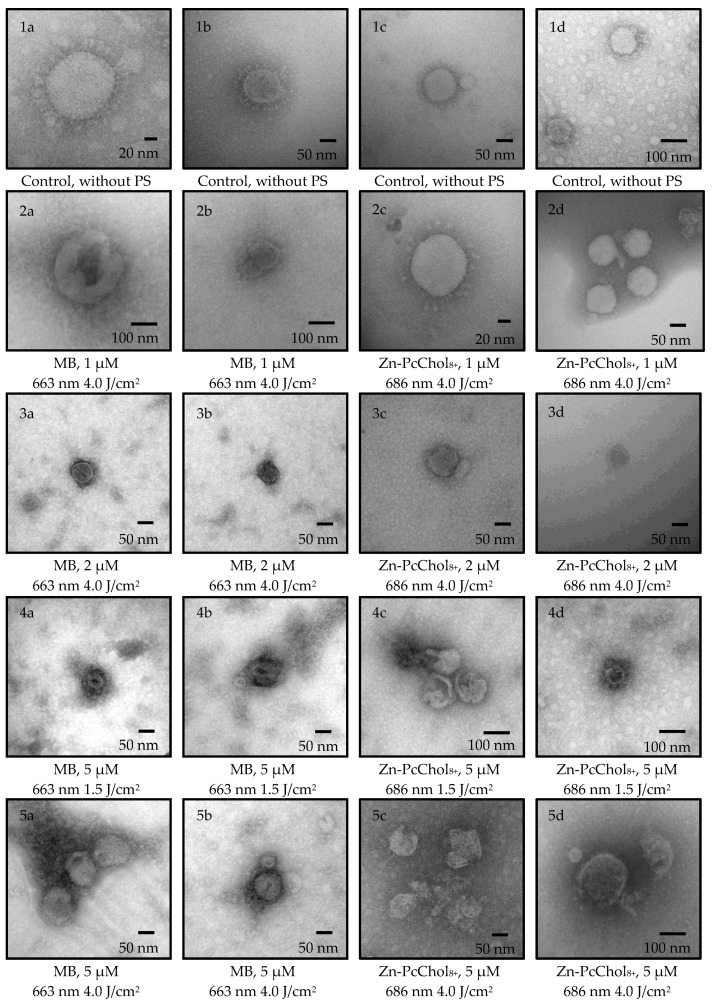
Electron micrographs of control BCoV, negatively stained with 2% uranyl acetate from Figure 2 (**1a**–**1d**). BCoV incubated at 10 min with 1 µM (**2a**–**2d**), 2 µM (**3a**–**3d**), or 5 µM (**4a**–**4d**,**5a**–**5d**) of MB or Zn-PcChol_8+_, and irradiated with LED 663 nm for MB or 686 nm for Zn-PcChol_8+_. Light doses were 1.5 J/cm^2^ (**4a**–**4d**) or 4.0 J/cm^2^ (**2a**–**2d**,**3a**–**3d**,**5a**–**5d**).

Photodynamic inactivation of BCoV was induced with a combined action of MB or Zn-PcChol_8+_ and appropriate light from LED source (Figure 3). Figure 4 shows the morphology of BCoV under various photodynamic exposure parameters (PS concentration and light dose).

After incubation with MB, which, even in the absence of irradiation, reduced the virus titer by three Logs, and irradiation with LED 663 nm caused a further reduction of about 1 Log_10_ TCID50/mL. In the presence of MB in the concentrations of 1–5 μM, envelope damage was characteristic of the photoinactivated viral particles (Figure 4(2a,2b,3a,3b,4a,4b,5a,5b)).

Along with the low “dark” antiviral activity of 1 μM Zn-PcChol_8+_, irradiation at 686 nm led to a 2 log decrease in virus titers. The efficiency of PDI with 2–5 μM Zn-PcChol_8+_ was almost the same with MB (Figure 3). With increasing concentrations of Zn-PcChol_8+_ (from 1 to 5 μM), and LED light with a maximum at 686 nm in the dose of 1.5–4.0 J/cm^2^, one can most clearly see the consistent appearance of the following morphological changes in the BCoV particles: the disappearance of spikes (Figure 4(2c,2d)), a change in the shape and size of viral particles (Figure 4(2c,2d,3c,3d)), destruction of the envelope (Figure 4(4c,4d)), and complete disintegration of viral structure (Figure 4(5c,5d)).

## 4. Discussion

In this study, we used two cationic dyes, MB and Zn-PcChol_8+_, to reduce titers of BCoV in vitro, and to study morphology of the inactivated virus particles. MB is the phenothiazinium dye with reported quantum yields of singlet oxygen production in different solvents at about 0.5 [31]. MB possesses dual antiviral activity. The first is the well-known MB light-induced activity which is used in THERAFLEX MB-Plasma (Macopharma) for plasma decontamination with visible light in the presence of MB [32,33]. Along with this, independently of light MB at very low micromolar concentrations, it is able to inhibit the replication of different viruses, including SARS-CoV-2 [25,33,34], Zika virus, Dengue virus [35], and the influenza virus H1N1 [34]. We also noted the non-photodynamic antiviral potential of MB. The short 10 min incubation with MB at concentrations of 1–5 μM reduced BCoV titers by 3 logs (Figure 3). The second dye, Zn-PcChol_8+_, also had the ability to inactivate BCoV in the absence of light, but at slightly higher concentrations of 2–5 µM compared with MB. Recently, “dark” virucidal activity has been shown in the tetrapyrrole Radachlorin [25] and phthalocyanine derivative [36]; however both dyes were anionic compounds, in contrast to the octacationic phthalocyanine derivative, Zn-PcChol_8+_, used in this study. The deformation and size reduction of virions in the presence of MB and Zn-PcChol_8+_ can be seen in Figure 2.

Electrostatic interactions play a key role in binding charged dye molecules to viral envelope structures. As we have shown recently [22], a significant negative charge in the S-proteins of coronaviruses is highly heterogeneously distributed on the protein surface. There are only a few areas of pronounced negative electrostatic potential that may attract cationic dyes, the foremost of which is common to at least three beta coronaviruses (SARS-CoV, SARS-CoV-2, MERS), and is at the connection of the S-protein stalk and the head adjacent to the HR2 domain [22]. In addition to this common site, MB is able to bind to the RBD domain of the SARS-CoV-2 S-protein [22]. This location of MB may prevent interaction of the S-proteins with the receptors on the host cell, thus reducing virus infectivity. In contrast to MB, Zn-PcChol_8+_ does not have binding sites at the RBD domain of the SARS-CoV-2 S-protein and lacks “dark” antiviral activity against SARS-CoV-2 [22,23]. The pronounced “dark” sensitivity of BCoV to Zn-PcChol_8+_, compared with its lack of “dark” sensitivity against SARS-CoV-2, may be associated with differences in the negative potential of their S-proteins and the presence in the BCoV envelope of not only the spike S-proteins, but also HE, the functional activity of which can be altered with Zn-PcChol_8+_. These suggestions need further research.

Antimicrobial photodynamic efficiency depends on many factors, such as the parameters of light exposure, the physicochemical and photochemical properties of PS, the presence of hosting structures on the surface of the pathogen suitable for PS binding, the composition of the medium that affects properties of PS and the binding process, the presence of microbial critical targets readily damaged with reactive oxygen species, and so on. Enveloped viruses provide a convenient target for the action of PSs. Proteins and unsaturated lipids of viral envelops are readily oxidized with reactive oxygen species and free radicals. Moreover, lipid peroxidation affects not only the properties of the membrane itself, but also the surrounding proteins [18].

In the electron micrographs of a negatively stained uranyl acetate herpes simplex virus-1, Smetana and coworkers [37] detected extensive photodynamic damage to the viral envelopes using the mixed sulfonated Pc-naphthalocyanine derivative and light. In influenza viruses treated with water suspension of C60 fullerene and light [38], or Zn-PcChol_8+_ and light [24], electron microscopy also revealed the destruction of the viral membrane. PDI with emodine containing PS PhytoQuin caused the emergence of spike proteins with abnormally high molecular weights in pseudotyped lentivirus, but it did not cause morphological changes in human coronavirus compared with non-irradiated samples. Despite this, the authors concluded that membranes were damaged based on data on the increased susceptibility of the genome in photosensitized HCoV-229E to degradation by ribonucleases [39]. In our study, photoactivated MB at a concentration of 1 μM caused damage to the viral membrane (Figure 4(2a,2b)); however, it was difficult to distinguish between morphological changes in BCoV caused by the virucidal action of MB itself or by photodynamic action. Spike detachment in BCoV treated with 1 μM of Zn-PcChol_8+_ and light (Figure 4(2c,2d)) can be attributed to the true photoinduced oxidative damage, as Zn-PcChol_8+_ at this concentration had low “dark” toxicity (Figure 3). Gross damage to the membrane of BCoV occurred with 5 μM of Zn-PcChol_8+_ and light (Figure 4(4c,4d,5c,5d)), and eventually led to the complete disintegration of the virus structure. A similar morphological change was noted with the appearance of “bald” viruses under less intense photodynamic exposure, and completely destroyed viruses under more intense ones, which we observed earlier during the photodynamic inactivation of the avian H5N8 influenza virus with the same PS [24]; however, unlike SARS-CoV-2 [23] and H5N8 [24], which completely lost infectivity when treated with the same concentrations of Zn-PcChol_8+_ and when irradiated, BCoV titers in most cases did not fall below 1 Log_10_ TCID50/mL, and only in some samples of BCoV with MB, did we observe total loss of virus infectivity as a result of photoinactivation.

## 5. Conclusions

Finally, MB (1–5 μM) and Zn-PcChol_8+_ (2–5 μM), in combination with irradiation in low doses of up to 4.0 J/cm^2^, obtained from the designated LED light sources, the spectrum of which corresponds to the absorption spectra of PSs, induce a reduction of BCoV titers by at least four orders of magnitude from the initial titer 5 Log_10_ TCID50/mL. Both dyes exhibit certain “dark” antiviral activity. MB and Zn-PcChol_8+_ are effective antimicrobial compounds that, in combination with appropriate irradiation, can be used as photodisinfectants. The results obtained expand the spectrum of viruses that can be inactivated using this technique. The fact that BCoV has been found to be sensitive to MB, even in the absence of irradiation, opens up the prospect of combating this pathogen which infects farm animals and causes serious economic damage, using this well-known, approved, phenothiazine drug.
